# SORTING NEXIN 1 Functions in Plant Salt Stress Tolerance Through Changes of NO Accumulation by Regulating NO Synthase-Like Activity

**DOI:** 10.3389/fpls.2018.01634

**Published:** 2018-11-06

**Authors:** Ting-Ting Li, Wen-Cheng Liu, Fang-Fang Wang, Qi-Bin Ma, Ying-Tang Lu, Ting-Ting Yuan

**Affiliations:** State Key Laboratory of Hybrid Rice, College of Life Sciences, Wuhan University, Wuhan, China

**Keywords:** SNX1, NO, NOS-like activity, salt stress, ROS

## Abstract

Nitric oxide (NO) production via NO synthase (NOS) plays a vital role in plant tolerance to salt stress. However, the factor(s) regulating NOS-like activity in plant salt stress tolerance remains elusive. Here, we show that *Arabidopsis SORTING NEXIN 1* (*SNX1*), which can restore H_2_O_2_-induced NO accumulation in yeast Δ*snx4* mutant, functions in plant salt stress tolerance. Salt stress induced NO accumulation through promoted NOS-like activity in the wild type, but this induction was repressed in salt-stressed *snx1-2* mutant with the mutation of *SNX1* because NOS-like activity was inhibited in the mutant. Consistently, *snx1-2* displayed reduced tolerance to high salinity with decreased survival rate compared with the wild type, and exogenous treatment with NO donor significantly rescued the hypersensitivity of the mutant to salt stress. In addition, the *snx1-2* mutant with reduced NOS-like activity repressed the expression of stress-responsive genes, decreased proline accumulation and anti-oxidant ability compared with wild-type plants when subjected to salt stress. Taken together with our finding that salt induces the expression of SNX1, our results reveal that SNX1 plays a crucial role in plant salt stress tolerance by regulating NOS-like activity and thus NO accumulation.

## Introduction

High salinity severely affects plant growth and cause substantial crop losses, posing a serious threat to global food security (Munns and Tester, 2008; Liu W. et al., 2015). Due to the sessile lifestyle, plants cannot avoid salt stress-triggered damage by changing their location. Therefore, plants have evolved sophisticated response mechanisms by perceiving the external sodium ion (Na^+^) concentration and optimizing adaptive responses to enhance their tolerance to salt stress (Park et al., 2016; Shi et al., 2017).

While the Salt Overly Sensitive (SOS) pathway has been well studied and recognized as an important mechanism controlling sodium ion homeostasis under salinity stress by increasing Na^+^ efflux (Lin et al., 2009; Yang and Guo, 2018), other protective mechanisms including regulating the expression of stress-responsive genes, accumulating proline and scavenging ROS also play crucial roles in plant salt stress tolerance (Khedr et al., 2003; Das and Roychoudhury, 2014). Besides, phytohormones also play roles in plant response and tolerance to salt stress (Xiong et al., 2002; Raghavendra et al., 2010). ABA, a well-known stress phytohormone induced by high salt stress, upregulates a large number of salt stress-responsive genes with various protective functions in tolerance (Fujita et al., 2009; Yoshida et al., 2010). Other phytohormones such as indole-3-acetic acid (IAA), gibberellic acid (GA) and ethylene are also involved in plant salt stress response by coordinating plant growth, development and stress tolerance (Achard et al., 2006; Liu W. et al., 2015; Ryu and Cho, 2015; Shi et al., 2017).

Nitric oxide (NO) as an important signaling molecule is also involved in diverse plant developmental processes and environmental stress responses such as high salinity (Grun et al., 2006; Del Rio, 2015). Exogenous treatment with NO donor, sodium nitroprusside (SNP), can enhance plant tolerance to salt stress by alleviating salt stress-induced oxidative damage and increasing Na^+^ efflux while reducing NO content by applying NO scavenger 2-(4-carboxyphenyl)-4,4,5,5- tetramethylimidazoline-1-oxyl-3-oxide (cPTIO) could severely reduce plant survival, demonstrating that NO plays an important role in plant salt stress response and tolerance (Shi et al., 2007; Ding, 2013; Dong et al., 2014; Ahmad et al., 2016; Jian et al., 2016). Further, high salt strongly induces NO accumulation in plant tissues by promoting NO synthase (NOS)-like activity (Fernandez-Marcos et al., 2011; Liu W. et al., 2015). While increasing NO accumulation by overexpressing rat neuronal NO synthase (*nNOS*) significantly enhances plant tolerance to salt stress (Shi et al., 2012; Cai et al., 2015), both NOS inhibitor-treated wild type and the (*Nitric Oxide Associated 1*) *noa1* mutant with reduced NOS-like activity and low NO accumulation displays hypersensitivity to high salinity (Zhao et al., 2007; Lozano-Juste and Leon, 2010; Xie et al., 2013), revealing the essential role of NOS-like activity in plant salt tolerance. However, how the NOS-like activity is modulated in plant salt stress response is unclear.

In this study, we report that *Arabidopsis SORTING NEXIN 1* (*SNX1*) has a novel role in regulating NOS-like activity and thus salt-induced NO accumulation in plant stress tolerance. SNX1, belongs to the sorting nexins family, is a part of retromer-like protein complex (Jaillais et al., 2006). It is colocalized with multivesicular body (MVB) markers in *Arabidopsis* root meristems, retrieving PIN proteins from a late/pre-vacuolar compartment back to the recycling pathways (Jaillais et al., 2006; Kleine-Vehn et al., 2008). Further researches reveal that SNX1 also localizes at the trans-Golgi network (TGN) (Niemes et al., 2010; Stierhof et al., 2013). SNX1 has various functions in plant growth and development, especially in responses to multiple environmental stimuli (Brumbarova and Ivanov, 2016; Heucken and Ivanov, 2018; Ivanov and Robinson, 2018). For example, SNX1 regulates plant response to high temperature by modulating auxin homeostasisis through PIN2 protein recycling (Hanzawa et al., 2013). Further study showed that GA could redirect PIN2 protein trafficking from the vacuolar pathway to the PM via SNX1-dependent protein retrieval (Salanenka et al., 2018). Also, SNX1 plays a role in iron homeostasis in plants upon iron deficiency by modulating the recycling of the iron transporter IRT1 (Blum et al., 2014; Ivanov et al., 2014). The functions of SNX1-mediated protein sorting in responses to environmental stimuli may be influenced by SNX1-interacting proteins, which can be regulated at transcriptional or post-transcriptional level (Brumbarova and Ivanov, 2016).

Our results show that salt-induced NOS-like activity and NO accumulation were compromised in *snx1-2* with the mutation of *SNX1* compared with those in the wild type, and thus the mutant showed hypersensitivity to salt stress. Exogenous application with NO donor sodium nitroprusside (SNP) or S-nitrosoglutathione (GSNO) can significantly rescue the reduced tolerance of *snx1-2* to high salinity. Furthermore, the *snx1-2* mutant with reduced NOS-like activity repressed the expression of stress-responsive genes, decreased proline accumulation and anti-oxidant ability. Taken together, SNX1 acts in plant salt stress tolerance through changes of NO accumulation by modulating NOS-like activity in Arabidopsis.

## Materials and Methods

### Strains, Media, and Treatments

The yeast *Saccharomyces cerevisiae* (*S. cerevisiae*) wild-type strain BY4741 (*MATα; his3*Δ*1; leu2*Δ*0; met*Δ*0; ura3*Δ*0*) and the deletion mutant Δ*snx4* (*YJL036W::kanMX4*) were purchased from *EUROSCARF* (Frankfurt, Germany).

For H_2_O_2_ treatment, yeast cells were grown until the early stationary growth phase in liquid YPD medium containing glucose (2%, w/v), yeast extract (0.5%, w/v) and peptone (1%, w/v). Cells were harvested and suspended (10^7^ cells/mL) in fresh YPD medium followed by the addition of 4 mM H_2_O_2_, then incubated for 30 min at 26°C with stirring (150 r.p.m.) as previously described (Almeida et al., 2007).

### Plasmid Construction and Transformation

The full length coding sequence of *Arabidopsis SNX1* was amplified using PCR and inserted into pYES260 vector at *NcoI* site behind the GAL1 promoter, resulting in *pYES260-SNX1*. The plasmid was then introduced into yeast mutant Δ*snx4* according to our previously reported method (Liu et al., 2017). Transformed cells were selected on solid SD medium (plus histidine, leucine, methionine and 1% galactose, without uracil and glucose). The *SNX1* specific primers used are listed in (Supplementary Table S1).

### Plant Materials and Growth Conditions

*Arabidopsis thaliana* ecotype Columbia was used in this study. *snx1-2* (T-DNA mutant, SALK_033351) and *SNX1::SNX1-mRFP* transgenic line were described in previous report (Jaillais et al., 2006). *Arabidopsis* seeds were surface sterilized for 5 min with 5% (w/v) bleach, washed three times with sterile water, incubated for 3 days at 4°C in the dark, and plated onto 1/2 MS agar medium (Sigma-Aldrich) 1% (w/v) Sucrose. Seedlings were grown in a growth chamber maintained at 23°C, 100 μmol m^-2^s^-1^ light under a 16 h-light/8 h-dark cycle.

### NO Detection

Free intracellular NO content was detected with the NO specific fluorescence dye DAF-FM DA (diaminofluorescein-FM diacetate, Sigma).

For NO detection in yeast cell, treated or untreated yeast cells were incubated in 50 mM potassium phosphate buffer (pH7.4) with 5 μM DAF-FM DA in the dark for 30 min. Then the yeast cells were rinsed and suspended in potassium phosphate buffer. For the visualization of NO florescence in yeast, the fluorescence images were taken under the fluorescence microscope (BX60, Olympus) equipped with a charge-coupled device (CCD) camera (Liu W.C. et al., 2015; Liu et al., 2017). NO content was represented by the fluorescence brightness, and the relative accumulation of NO was expressed in units of luminance (AU) in the Photograph histogram.

For NO detection in *Arabidopsis* roots, seedlings treated with or without 100 mM NaCl for 24 h were incubated in 50 mM potassium phosphate buffer (pH7.4) with 5 μM DAF-FM DA in the dark for 30 min. Then the seedlings were rinsed and suspended in potassium phosphate buffer. For the visualization of NO florescence in roots, the fluorescence images were taken under the fluorescence microscope (BX60, Olympus) equipped with a charge-coupled device (CCD) camera (Liu W.C. et al., 2015; Liu et al., 2017). NO content was represented by the fluorescence brightness, and the relative accumulation of NO was expressed in units of luminance (AU) in the Photograph histogram.

### Measurement of NOS-Like Activity

For NOS-like activity detection in plants, treated or untreated *Arabidopsis* seedlings were ground with liquid nitrogen and then resuspended in the extraction buffer (50 mM Tris-HCl, pH 7.4, 1 mM EDTA, 1 mM dithiothreitol, 1 mM leupeptin, 1 mM pepstatin, and 1 mM phenylmethylsulfonyl fluoride). The mixture was vortexed and centrifuged at 12,000 rpm for 15 min at 4°C. The supernatant was used for NOS activity determination with a NOS assay kit (Beyotime, Haimen, China) as previously described reports (Shi et al., 2012). Briefly, 0.1 mL supernatant was added into 0.1 mL reactio mixture (containing NADPH, L-Arg, NOS assay buffer and DAF-FM DA) and reacted at 37°C in the dark for 1 h. The production of NO was measured using a fluoremeter with 488 nm excitation and 510 nm emission filters.

### RNA Extraction and Expression Analysis

RNA extraction and quantitative real-time PCR (qRT-PCR) were performed according to our previously described method (Liu et al., 2017; Yuan et al., 2017). Total RNA extraction was performed using PureLink^TM^ Plant RNA Reagent (Invitrogen) according to the manufacturer’s instruction. RNA samples were then treated with RQ1 RNase-free DNase I (Promega) to remove DNA. The reverse transcription was carried out by using ReverTra Ace^®^ (Toyobo). qRT-PCR assay was performed by using a CFX96^TM^ Real-Time PCR Detection System (Bio-Rad) with *ACT2/8* (AT3G18780, AT1G49240) as the reference gene. All experiments were performed with three independent biological replicates and three technical repetitions. The primers used are listed in Supplementary Table S1.

### Immunoblot Analysis

Total proteins extracted from 5 days old seedlings treated with or without 100 mM NaCl for 6 h were separated by 12% SDS–PAGE. Immunoblotting was carried out on PVDF membranes with anti-mRFP antibody (D110087, BBI Life Sciences). Coomassie Brilliant Blue staining (CBB) indicates equal total protein loading.

### NBT Staining

Superoxide free radicals were detected as described previously with minor modifications (Guan et al., 2013). Briefly, 5 days old seedlings grown on 1/2 MS medium were treated with or without 100 mM NaCl for 24 h, then the seedlings were vacuum-infiltrated with 0.1 mg/mL nitroblue tetrazolium (Sigma ^1^) in 25 mM HEPES buffer (pH 7.6) for 2 h in darkness. Chlorophyll was removed using 70% ethanol and then plants tissues were photographed.

### Measurement of Proline Content

Proline content in salt-treated or untreated seedlings was performed according to our previous reports with L-proline as the standard. In brief, proline content in *Arabidopsis* seedling was measured according to a previously described method (Zhu et al., 2016). About 0.5 g of *Arabidopsis* seedlings were ground into powder with liquid nitrogen and extracted in 3% sulfosalicylic acid. After centrifuging at 12,000 g for 10 min, the supernatant (2 mL) was mixed with 2 mL of ninhydrin reagent (2.5% (w/v) ninhydrin, 60% (v/v) glacial acetic acid, 40% 6 M phosphoric acid) and 2 mL of glacial acetic acid. After incubation at 100°C for 40 min, the reaction was terminated in an ice bath. Then 5 mL toluene was added, followed by vortex. Finally, the absorbance was measured at 520 nm with a UV-5200 spectrophotometer.

### Confocal Microscopy

Confocal microscopy was performed using a FluoView 1000 Confocal Laser-scanning Microscope according to the manufactuer’s instructions and our previously described method (Liu W.C. et al., 2015). mRFP was excited with 561 nm, and its emissions were detected between 580 and 620 nm. Two objectives (20× and 40×) were used for magnification micrographs. Twelve seedlings treated with or without 100 mM NaCl for 6 h were analyzed. The signal intensity was analyzed using Photoshop CS5 (Adobe, San Jose, CA, United States).

## Results

### Yeast SNX4 and *Arabidopsis* SNX1 Conservatively Act in the Regulation of NO Accumulation

**FIGURE 1:**
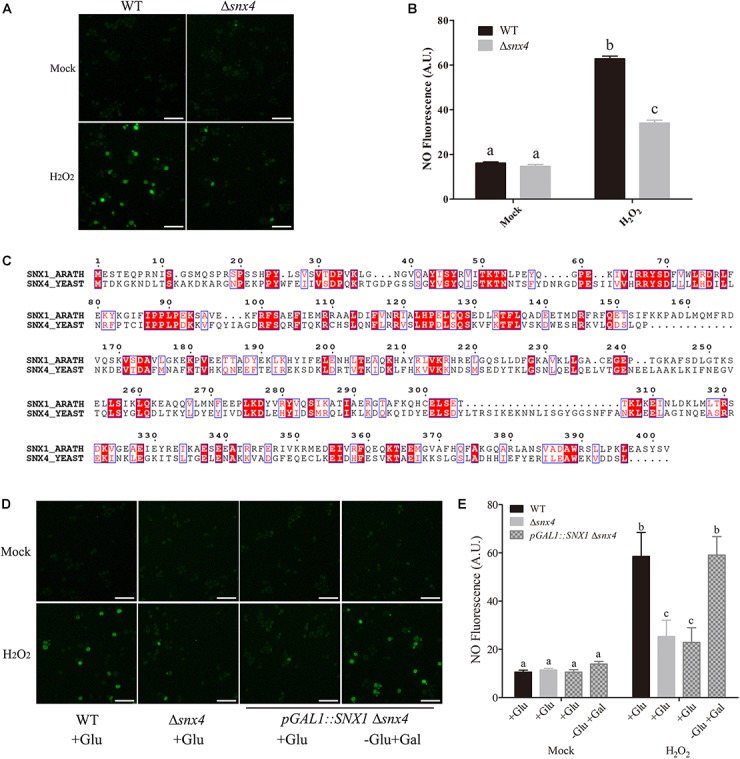
Yeast SNX4 and *Arabidopsis* SNX1 conservatively act in the regulation of NO synthesis. **(A)** NO production was shown by NO-specific dye DAF-FM DA fluorescence staining in wild type yeast and Δ*snx4* treated with or without 4 mM H_2_O_2_ for 30 min. Scale bars = 50 μm. **(B)** NO content in **(A)** was expressed using the fluorescence from DAF-FM DA staining. DAF-FM DA fluorescence is indicated as pixel intensity arbitrary units (AU). Data shown are means ± SEM. Different letters indicate significant differences between treatments (*P* < 0.05 by one-way ANOVA with Tukey’s multiple comparison test). **(C)** Protein sequences alignment of *Arabidopsis* SNX1 and Yeast SNX4. The identical amino acid residues are colored with red. **(D)** The wild-type yeast, Δ*snx4* and *pGAL::SNX1*Δ*snx4* cells were cultured in SD medium (+Glu), and *pGAL::SNX1*Δ*snx4* cells were induced with SD medium (-Glu + Gal). NO production was shown by DAF-FM DA staining in the indicated yeast cells with or without 4 mM H_2_O_2_ treatment for 30 min. Scale bars = 50 μm. **(E)** NO content in **(D)** was expressed using the fluorescence from DAF-FM DA staining. DAF-FM DA fluorescence is indicated as pixel intensity arbitrary units (AU). Data shown are means ± SEM. Different letters indicate significant differences between treatments (*P* < 0.05 by one-way ANOVA with Tukey’s multiple comparison test).

NOS-dependent NO synthesis plays a vital role in plant response and tolerance to salt stress, whereas the factor(s) regulating NOS-like activity in plant salt stress response is unknown. Our previous study identified several genes probably involved in the regulation of NOS-like activity in yeast when treated with H_2_O_2_ by screening for the mutants with lower NO accumulation from a collection of about 7800 yeast deletion mutants because H_2_O_2_ activates NOS-dependent NO accumulation (Almeida et al., 2007). Here, we focused on one of these yeast mutants, Δ*snx4* (YJL036W) with the mutation of *Sorting Nexin 4* (*SNX4*). NO accumulation in Δ*snx4* mutant was much lower than that in wild-type cells upon H_2_O_2_ exposure (Figures 1A,B), revealing the involvement of *SNX4* in the regulation of H_2_O_2_-induced NO accumulation in yeast.

To search for the homolog of yeast SNX4, the identified *Arabidopsis* SNX1 which shares 25% identity with yeast SNX4 had been found (Figure 1C). This plant protein is a part of a retromer-like protein complex and involved in endosome to lysosome protein transport (Jaillais et al., 2006; Ambrose et al., 2013; Ivanov et al., 2014). However, the role of SNX1 in the regulation of NO accumulation in plants remains unknown. To assess whether SNX1 plays a role in the regulation of NO accumulation as yeast SNX4, we transformed *pYES260-AtSNX1* plasmid into Δ*snx4*, where the expression of *AtSNX1* is driven by galactose-induced yeast *GAL1* promoter. When subjected to H_2_O_2_ treatment, the reduced NO accumulation in Δ*snx4* is rescued by *AtSNX1* expression in *pGAL1-AtSNX1*Δ*snx4* in the presence of galactose (Figures 1D,E), demonstrating that *Arabidopsis* SNX1 and yeast SNX4 play a conserved role in H_2_O_2_-induced NO accumulation.

### *Arabidopsis* SNX1 Functions in Salt Stress Tolerance by Regulating NOS-Like Activity and Thus NO Accumulation

**FIGURE 2:**
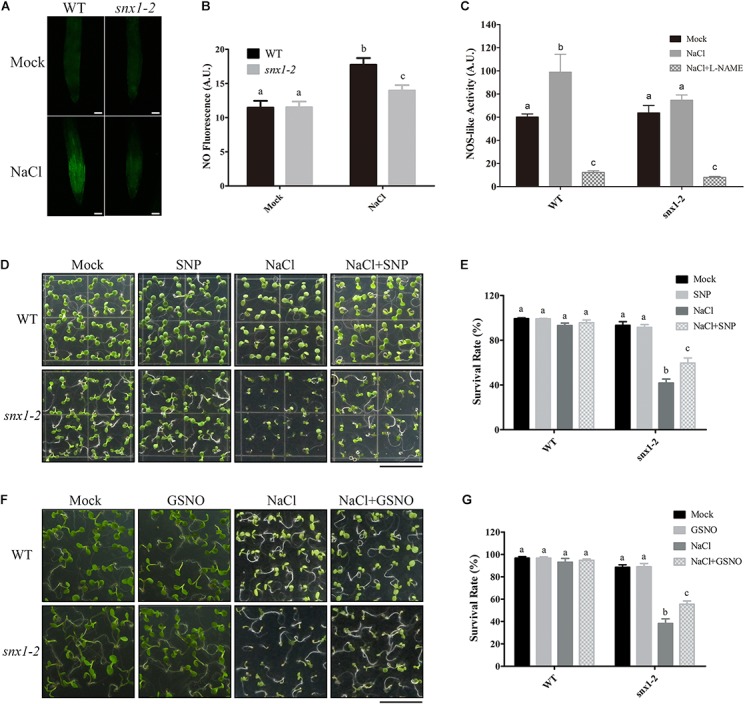
*Arabidopsis SNX1* functions in salt stress tolerance by regulating NOS-like activity and NO accumulation. **(A)** NO accumulation was shown by DAF-FM DA staining in wild-type and *snx1-2* mutant seedlings with or without 100 mM NaCl treatment for 24 h. Scale bars = 50 μm. **(B)** NO content in **(A)** was expressed using the fluorescence from DAF-FM DA staining. DAF-FM DA fluorescence is indicated as pixel intensity arbitrary unit(AU). At least 12 seedlings were imaged per treatment for each of the three replicates. Data shown are means ± SEM. Different letters indicate significant differences between treatments (*P* < 0.05 by one-way ANOVA with Tukey’s multiple comparison test). **(C)** The wild-type and *snx1-2* mutant plants were treated with 100 mM NaCl for 24 h in the presence of 1 mM L-NAME or not, and then assayed NOS-like activity. Data shown are means ± SEM.Different letters indicate significant differences between treatments (*P* < 0.05 by one-way ANOVA with Tukey’smultiple comparison test). **(D)** Survival rate of the wild-type and *snx1-2* mutant plants in the present or absence of SNP under salt stress. Survival phenotypes of the wild-type and *snx1-2* mutant seedlings grown on 1/2MS medium containing 5 μM SNP, 125 mM NaCl or 5 μM SNP + 125 mM NaCl. Scale bars = 1cm. **(E)** Survival rate analysis in **(D)**. At least 100 seedlings were counted per treatment for each of the three replicates. Data shown are means ± SEM. Different letters indicate significant differences between treatments (*P* < 0.05 by one-way ANOVA with Tukey’s multiple comparison test). **(F)** Survival rate of the wild-type and *snx1-2* mutant plants in the present or absence of GSNO under salt stress. Survival phenotypes of the wild-type and *snx1-2* mutant seedlings grown on 1/2MS medium containing 50 μM GSNO, 125 mM NaCl or 50 μM GSNO + 125 mM NaCl. Scale bars = 1 cm. **(G)** Survival rate analysis in **(F)**. At least 100 seedlings were counted per treatment for each of the three replicates. Data shown are means ± SEM. Different letters indicate significant differences between treatments (*P* < 0.05 by one-way ANOVA with Tukey’s multiple comparison test).

Since NO plays a vital role in plant response and tolerance to high salinity and SNX1 modulate NO accumulation in yeast, we investigated whether SNX1 affects salt stress-induced NO accumulation in plant salt stress response with *snx1-2*. The *snx1-2*, a T-DNA insertion null mutant of SNX1, has been used to indicate its role in modulating PIN2 endosomal transport in pleiotropic auxin related defects (Jaillais et al., 2006). We examined NO accumulation in the roots of wild-type and *snx1-2* seedlings treated with salt stress. Our results showed that salt stress significantly induced NO accumulation in the roots of wild-type seedling as previously reported (Liu W. et al., 2015; Figures 2A,B). This induction of NO accumulation in *snx1-2* was repressed (Figures 2A,B), supporting that SNX1 functions in salt-induced NO accumulation in plants. This repression of NO accumulation is due to the inhibited NOS-like activity in *snx1-2* because *snx1-2* seedlings had lower NOS-like activity than wild-type plants and increased NOS-like activity in wild-type plants was inhibited by NOS specific inhibitor, L-NAME when challenged with high salt (Figure 2C).

To further assess whether changes in NO accumulation in *snx1-2* affect plant salt stress tolerance, we assayed the sensitivity of *snx1-2* to high salinity in terms of survival rate of the salt-stressed mutant. The survival rate of *snx1-2* was much lower than that in the wild type after salt stress treatment (Figures 2D–G), suggesting that the reduced NO accumulation of *snx1-2* results in its hypersensitivity to salt stress. To further support it, we examined whether NO donor can rescue the reduced survival rate of *snx1-2* compared with the wild type treated with high salinity. We found that both SNP and GSNO can significantly enhance the survival rate of *snx1-2* compared with untreated control (Figures 2D–G). Taken together, *Arabidopsis* SNX1 functions in salt stress tolerance by change of NO accumulation through its regulation of NOS-like activity.

### *SNX1* Acts in Plant Salt Stress Tolerance by Regulating the Expression of Salt-Responsive Genes and Proline Synthesis

**FIGURE 3:**
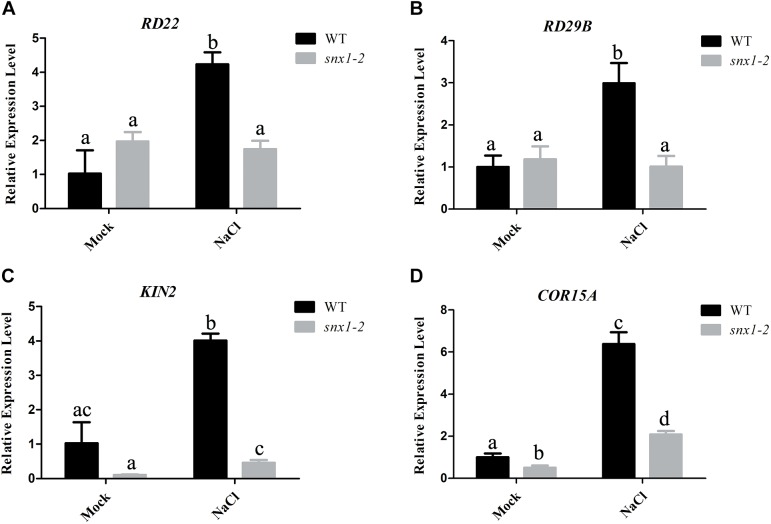
Expression of salt-responsive genes in the wild type and *snx1-2* plants treated with or without salt stress. **(A–D)** Five days old wild-type and *snx1-2* mutant seedings were treated with 100 mM NaCl for 24 h, and then assayed the expression of salt-responsive genes *RD22* **(A)**, *RD29B* **(B)**, *KIN2* **(C)**, *COR15A* **(D)**. Data shown are means ± SEM. Different letters indicate significant differences between treatments (*P* < 0.05 by one-way ANOVA with Tukey’s multiple comparison test).

**FIGURE 4:**
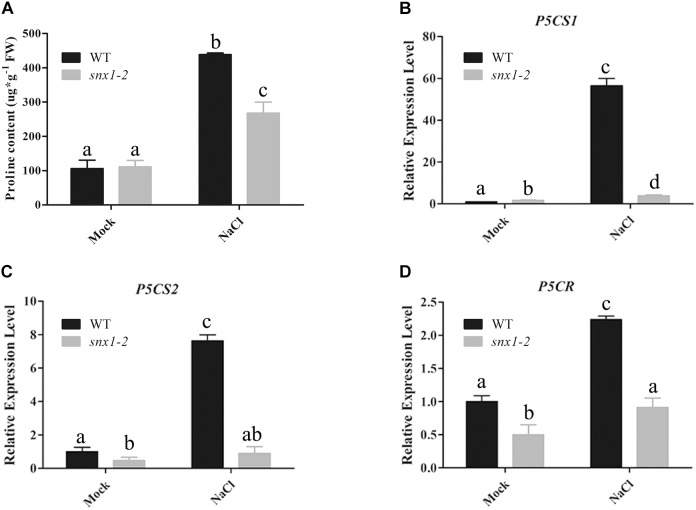
Proline content and expression of proline synthetic genes in the wild-type and *snx1-2* mutant seedlings treated with or without salt stress. **(A)** Proline content in wild type and *snx1-2* mutant seedlings under salt stress. Five days old seedlings of wild type *Arabidopsis thaliana* or *snx1-2* mutant were treated with 125 mM NaCl for 3 days or not, and assayed the proline content in seedlings. Data shown are means ± SEM. Different letters indicate significant differences between treatments (*P* < 0.05 by one-way ANOVA with Tukey’s multiple comparison test). **(B–D)** Expression of proline synthetic genes in the wild type and *snx1-2* mutant seedlings treated with or without 100 mM NaCl treatment for 24 h *P5CS1* **(B)**, *P5CS2* **(C)**, *P5CR* **(D)**. Data shown are means ± SEM. Different letters indicate significant differences between treatments (*P* < 0.05 by one-way ANOVA with Tukey’s multiple comparison test).

It is well-known that NO plays its role in environmental stresses including salt or drought stress by activating the expression of many stress-responsive genes and promoting proline accumulation (Shi et al., 2012; Cai et al., 2015). Thus, we also examined whether changes of NO accumulation in *snx1-2* with reduced survival rate affect the expression of stress-responsive genes by RT-qPCR. When subjected to high salinity. The stress-responsive genes such as *RD22*, *RD29B*, *KIN2*, and *COR15A* were significantly induced by salt stress in the wild type, but the increased expression of these genes were repressed in salt-treated *snx1-2* seedlings (Figures 3A–D). Similarly, the increased proline accumulation in the wild type was also reduced in *snx1-2* when subjected to salt stress (Figure 4A). Reduced proline content could be due to decreased expression of proline biosynthetic genes, thus we further investigated the expression of *P5CR1*, *P5CS2*, and *P5CR* involved in proline biosynthesis. Indeed, when challenged with high salt, the expressions of these genes were significantly induced in wild type but this induction was suppressed *snx1-2* (Figures 4B–D). These results suggest that SNX1 acts in plant salt stress tolerance by regulating the expression of salt-responsive genes and proline biosynthesis.

### SNX1 Modulates ROS Homeostasis in Plant Tolerance to Salt Stress

**FIGURE 5:**
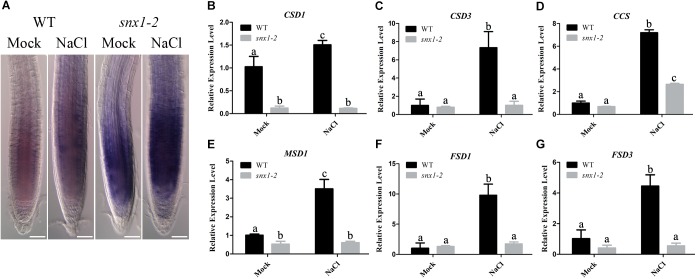
SNX1 modulates ROS homeostasis in plant tolerance to salt stress. **(A)** The wild-type and *snx1-2* seedlings were treated with 100 mM NaCl for 24 h, and then superoxide anions were visualized NBT staining. At least 12 seedlings were imaged per treatment for each of the three replicates. Scale bars = 50 μm. **(B–G)** Five days old seedlings of wild type and *snx1-2* mutant were treated with or without 100 mM NaCl for 24 h, and then the expression of ROS-scavenging genes were assayed. Data shown are means ± SEM. Different letters indicate significant differences between treatments (*P* < 0.05 by one-way ANOVA with Tukey’s multiple comparison test).

Salt stress causes production of reactive oxygen species (ROS) including superoxide anion, causing oxidative damage to plant cells (Apel and Hirt, 2004; Foyer and Noctor, 2005; Das and Roychoudhury, 2014; Del Rio, 2015). Thus, we explored whether *snx1-2* mutant with reduced NO accumulation has higher accumulation of superoxide anion, leading to sensitivity to salt stress. For this purpose, we assayed the accumulation of superoxide anion in wild-type and *snx1-2* seedlings treated with high salinity using nitrotetrazolium blue (NBT) staining. Our results showed that NBT staining in the roots of *snx1-2* seedlings were darker than that in the wild type, either with or without salt stress treatment (Figure 5A), indicating that the superoxide anion accumulation is higher in *snx1-2*. Then, we examined the expression of genes encoding superoxide dismutase (SOD), which can detoxify superoxide radicals. We found that all tested *SOD* genes (*CSD1*, *CSD3*, *CCS*, *MSD1*, *FSD1*, and *FSD2*) were induced by salt stress in the wild type, but this induction were significantly repressed in *snx1-2*, indicating a role of SNX1 in regulating superoxide accumulation by changes of *SODs* expression in plant salt stress tolerance (Figures 5B–G). These data indicates that SNX1 plays an important role in modulating ROS homeostasis in plant tolerance to salt stress.

### Salt Induces the Expression of SNX1

**FIGURE 6:**
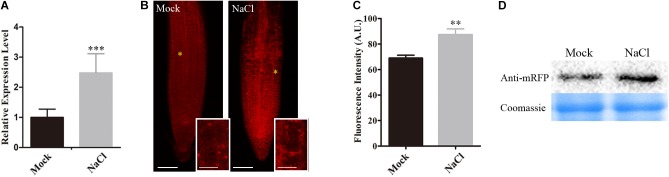
Salt induces the expression of SNX1. **(A)** The wild-type seedlings were treated with or without 100 mM NaCl for 6 h, and then *SNX1* expression was assayed. **(B)** Five days old seedlings of *SNX1::SNX1-mRFP* treated with or without 100 mM NaCl for 6 h were used to assay the fluorescence of mRFP. Scale bars = 50 μm. High magnification micrographs of the area outline with a yellow asterisks in low magnification micrographs. Scale bars = 10 μm. **(C)** Quantified fluorescence intensities in **(B)**. At least 12 seedlings were imaged per treatment for each of the three replicates. Data shown are means ± SEM. Asterisks indicate significant differences with respect to each control (Student’s *t*-test, ^∗^*p* < 0.05, ^∗∗^*p* < 0.01, ^∗∗∗^*p* < 0.001). **(D)** SNX1 Protein in 5 days old *SNX1::SNX1-mRFP* seedlings treated with or without 100 mM NaCl for 6 h were assayed by western blot with anti-mRFP antibody.

Our above results showed that SNX1 is a novel factor regulating salt stress tolerance through changes of NO accumulation in plants, we further explored whether the expression of *SNX1* was influenced by salt stress. Our qRT-PCR analyses indicated that the expression of *SNX1* was significantly induced by salt (Figure 6A). We also assessed the accumulation of SNX1 protein in plants challenged with high salinity using *SNX1::SNX1-mRFP* transgenic plant as previously reported (Jaillais et al., 2006). We found that RFP fluorescence in the roots of salt-treated *SNX1::SNX1-mRFP* seedlings was higher than that in untreated control (Figures 6B,C). Consistently, higher *SNX1-mRFP* protein accumulation was detected in salt-treated *SNX1::SNX1-mRFP* seedlings (Figure 6D). These results indicate that plants increase SNX1 protein accumulation in response to salt stress.

Taken together, our results revealed that plant upregulates SNX1 to modulate NO accumulation through changes of NOS-like activity in response to salt stress.

## Discussion

As an important bioactive molecule, the biosynthesis of NO has always been a concern. In mammals, NO is synthesized by NOS with L-Arg as substrate (Gupta et al., 2011). In plants, NOS and nitrate reductase (NR) have been known as two major sources of NO production (Wilson et al., 2008; Gupta et al., 2011). However, the gene encoding NOS in plants has not been identified so far, and thus the mechanism underlying the regulation of NOS-like activity is poorly understood. To search for new factors involved in modulating NOS-like activity in Arabidopsis, we carried out the experiment to screen for possible players in yeast first, and then identify their homologs in Arabidopsis. Using this method, we show that expression of *Arabidopsis SNX1* restores H_2_O_2_-indcued NO accumulation in yeast mutant Δ*snx4*, indicating that *Arabidopsis SNX1* and yeast *SNX4* are homologous genes and play a conserved role in regulating NO accumulation.

In yeast, SNX4 has been reported to involve in the retrieval of late-Golgi from post-Golgi endosomes to the trans-Golgi network and the transport from cytoplasm to vacuole in yeast (Hettema et al., 2003; Bean et al., 2017). In Arabidopsis, SNX1, which has 25% identity with yeast SNX4 protein sequence, participates in endosome to lysosome protein transport (Ambrose et al., 2013; Ivanov et al., 2014).

In this study, we uncover a novel role of SNX1 in NO accumulation by changes of NOS-like activity. But, how SNX1 modulates NOS-like activity remains unknown. The SNX1 may affect NOS-like activity through its interaction with NOS. However, we could not identify any of these proteins to be NOS candidate. A previous report demonstrates that NOA1 functions as a modulator of NOS-like activity that mediates changes in NO accumulation (Guo et al., 2003). Recently, WD40-REPEAT 5a has been reported to regulate NOS-like activity with unknown mechanism (Liu et al., 2017). Therefore, we further speculate that SNX1 may affect NOS-like activity as a secondary effect, by interacting with and affecting the direct NOS regulators. The stress-responsive genes, proline biosynthetic genes and *SOD* genes were significantly induced by salt stress in the wild type, but the increased expression of these genes was repressed in salt-treated *snx1-2* seedlings (Figures 3–5). These results suggest that SNX1 acts in plant salt stress tolerance by regulating the expression of salt-responsive genes, proline biosynthesis genes and *SOD* genes. Thus, we further investigated whether the expression of these genes induce by NO. When treated with NO donor SNP, *RD22*, *KIN2*, *COR15A*, *P5CS1*, *P5CS2*, and *P5CR* were significantly induced by SNP in both wild-type and *snx1-2* seedlings (Supplementary Figures S1A–D, S2A–C), suggesting that these genes could induced by SNP. These results were consistent with previous studies that many stress-response genes are regulated by NO (Shi et al., 2012; Hussain et al., 2016). However, we found that SNP could not enhance the expression of *SOD* genes in both wild-type and *snx1-2* seedlings (Supplementary Figures S3A–F), suggesting SNP could not affect the expression of *SOD* genes.

As a key signaling molecule, NO plays essential roles in various plant physiological and developmental processes as well as plant responses to biotic and abiotic stresses, including salt stress. High salinity strongly induces NO accumulation in plant tissues (Fernandez-Marcos et al., 2011; Liu W. et al., 2015), and NOS-dependent NO synthesis plays an essential role in plant tolerance to high salinity (Zhao et al., 2007; Lozano-Juste and Leon, 2010; Shi et al., 2012; Xie et al., 2013; Cai et al., 2015). However, how the NOS-like activity is modulated in plant salt stress response is unclear. Here, we show that the *snx1-2* mutant with lower NOS-like activity and thereby less NO accumulation is hypersensitive to high salinity. Since NO is involved in diverse plant developmental processes and environmental responses, whether *SNX1* also acts in such processes and responses is, therefore, worthy of further exploration.

While NO donor can significantly enhance the survival rate of *snx1-2* mutant compared with untreated control, NO donor cannot completely rescue the reduced survival rate of *snx1-2* mutant compared with the wild type when treated with high salinity (Figures 2D–G), indicating that there is another mechanism that SNX1 functions in salt stress tolerance. It has been demonstrated that SNX1 interacts with sodium-proton exchanger 6 (NHX6), which could enhance salt tolerance in a variety of species (Ashnest et al., 2015). SNX1 may affect the salt tolerance though interacting with NHX6 and therefore enhance plant salt tolerance. In conclusion, our results reveal that SNX1 is a novel factor in plant salt stress tolerance through changes of NOS-like ability and thereby NO accumulation in Arabidopsis.

## Author Contributions

T-TL and T-TY conceived and designed the experiments. T-TL, W-CL and F-FW performed the experiments and analyzed the data. T-TY and Y-TL wrote the manuscript. All authors read and approved the final manuscript.

## Conflict of Interest Statement

The authors declare that the research was conducted in the absence of any commercial or financial relationships that could be construed as a potential conflict of interest.

## Footnotes

1 www.sigmaaldrich.com

## References

[B1] Achard P. Cheng H. De Grauwe L. Decat J. Schoutteten H. Moritz T. (2006). Integration of plant responses to environmentally activated phytohormonal signals. *Science* 311 91–94. 10.1126/science.1118642 16400150

[B2] Ahmad P. Latef A. A. A. Hashem A. Abd Allah E. F. Gucel S. Tran L. S. P. (2016). Nitric oxide mitigates salt stress by regulating levels of osmolytes and antioxidant enzymes in chickpea. *Front. Plant Sci.* 7:347. 10.3389/fpls.2016.00347 27066020 PMC4814448

[B3] Almeida B. Buttner S. Ohlmeier S. Silva A. Mesquita A. Sampaio-Marques B. (2007). NO-mediated apoptosis in yeast. *J. Cell Sci.* 120 3279–3288. 10.1242/jcs.010926 17726063

[B4] Ambrose C. Ruan Y. Gardiner J. Tamblyn L. M. Catching A. Kirik V. (2013). CLASP interacts with sorting nexin 1 to link microtubules and auxin transport via PIN2 recycling in *Arabidopsis thaliana*. *Dev. Cell* 24 649–659. 10.1016/j.devcel.2013.02.007 23477787

[B5] Apel K. Hirt H. (2004). Reactive oxygen species: metabolism, oxidative stress, and signal transduction. *Annu. Rev. Plant Biol.* 55 373–399. 10.1146/annurev.arplant.55.031903.141701 15377225

[B6] Ashnest J. R. Huynh D. L. Dragwidge J. M. Ford B. A. Gendall A. R. (2015). *Arabidopsis* intracellular NHX-Type sodium-proton antiporters are required for seed storage protein processing. *Plant Cell Physiol.* 56 2220–2233. 10.1093/pcp/pcv138 26416852

[B7] Bean B. D. M. Davey M. Conibear E. (2017). Cargo selectivity of yeast sorting nexins. *Traffic* 18 110–122. 10.1111/tra.12459 27883263

[B8] Blum A. Brumbarova T. Bauer P. Ivanov R. (2014). Hormone influence on the spatial regulation of IRT1 expression in iron-deficient *Arabidopsis thaliana* roots. *Plant Signal. Behav.* 9:28787. 10.4161/psb.28787 24721759 PMC4091473

[B9] Brumbarova T. Ivanov R. (2016). ”Differential gene expression and protein phosphorylation as factors regulating the state of the *Arabidopsis* SNX1 protein complexes in response to environmental stimuli. *Front. Plant Sci.* 7:1456. 10.3389/fpls.2016.01456 27725825 PMC5035748

[B10] Cai W. Liu W. Wang W. S. Fu Z. W. Han T. T. Lu Y. T. (2015). Overexpression of rat neurons nitric oxide synthase in rice enhances drought and salt tolerance. *PLoS One* 10:e0131599. 10.1371/journal.pone.0131599 26121399 PMC4485468

[B11] Das K. Roychoudhury A. (2014). Reactive oxygen species (ROS) and response of antioxidants as ROS-scavengers during environmental stress in plants. *Front. Environ. Sci.* 2:53 10.3389/fenvs.2014.00053

[B12] Del Rio L. A. (2015). ROS and RNS in plant physiology: an overview. *J. Exp. Bot.* 66 2827–2837. 10.1093/jxb/erv099 25873662

[B13] Ding F. (2013). Effects of salinity and nitric oxide donor sodium nitroprusside (SNP) on development and salt secretion of salt glands of *Limonium bicolor*. *Acta Physiol. Plant.* 35 741–747. 10.1007/s11738-012-11148

[B14] Dong Y. J. Jinc S. S. Liu S. Xu L. L. Kong J. (2014). Effects of exogenous nitric oxide on growth of cotton seedlings under NaCl stress. *J. Soil Sci. Plant Nutr.* 14 1–13. 10.4067/S0718-95162014005000001

[B15] Fernandez-Marcos M. Sanz L. Lewis D. R. Muday G. K. Lorenzo O. (2011). Nitric oxide causes root apical meristem defects and growth inhibition while reducing PIN-FORMED 1 (PIN1)-dependent acropetal auxin transport. *Proc. Natl. Acad. Sci. U.S.A.* 108 18506–18511. 10.1073/pnas.1108644108 22021439 PMC3215072

[B16] Foyer C. H. Noctor G. (2005). Redox homeostasis and antioxidant signaling: a metabolic interface between stress perception and physiological responses. *Plant Cell* 17 1866–1875. 10.1105/tpc.105.033589 15987996 PMC1167537

[B17] Fujita Y. Nakashima K. Yoshida T. Katagiri T. Kidokoro S. Kanamori N. (2009). Three SnRK2 protein kinases are the main positive regulators of abscisic acid signaling in response to water stress in *Arabidopsis*. *Plant Cell Physiol.* 50 2123–2132. 10.1093/pcp/pcp147 19880399

[B18] Grun S. Lindermayr C. Sell S. Durner J. (2006). Nitric oxide and gene regulation in plants. *J. Exp. Bot.* 57 507–516. 10.1093/jxb/erj053 16396997

[B19] Guan Q. M. Lu X. Y. Zeng H. T. Zhang Y. Y. Zhu J. H. (2013). Heat stress induction of miR398 triggers a regulatory loop that is critical for thermotolerance in *Arabidopsis*. *Plant J.* 74 840–851. 10.1111/tpj.12169 23480361

[B20] Guo F. Q. Okamoto M. Crawford N. M. (2003). Identification of a plant nitric oxide synthase gene involved in hormonal signaling. *Science* 302 100–103. 10.1126/science.1086770 14526079

[B21] Gupta K. J. Fernie A. R. Kaiser W. M. van Dongen J. T. (2011). On the origins of nitric oxide. *Trends Plant Sci.* 16 160–168. 10.1016/j.tplants.2010.11.007 21185769

[B22] Hanzawa T. Shibasaki K. Numata T. Kawamura Y. Gaude T. Rahman A. (2013). Cellular auxin homeostasis under high temperature is regulated through a sorting NEXIN1-dependent endosomal trafficking pathway. *Plant Cell* 25 3424–3433. 10.1105/tpc.113.115881 24003052 PMC3809541

[B23] Hettema E. H. Lewis M. J. Black M. W. Pelham H. R. B. (2003). Retromer and the sorting nexins Snx4/41/42 mediate distinct retrieval pathways from yeast endosomes. *EMBO J.* 22 548–557. 10.1093/Emboj/Cdg062 12554655 PMC140746

[B24] Heucken N. Ivanov R. (2018). The retromer, sorting nexins and the plant endomembrane protein trafficking. *J. Cell Sci.* 131:jcs203695. 10.1242/jcs.203695 29061884

[B25] Hussain A. Mun B. G. Imran Q. M. Lee S. U. Adamu T. A. Shahid M. (2016). Nitric Oxide mediated transcriptome Profiling reveals activation of multiple regulatory pathways in *Arabidopsis thaliana*. *Front. Plant Sci.* 7:975. 10.3389/fpls.2016.00975 27446194 PMC4926318

[B26] Ivanov R. Brumbarova T. Blum A. Jantke A. M. Fink-Straube C. Bauer P. (2014). SORTING NEXIN1 is required for modulating the trafficking and stability of the *Arabidopsis* iron-regulated transporter1. *Plant Cell* 26 1294–1307. 10.1105/tpc.113.116244 24596241 PMC4001385

[B27] Ivanov R. Robinson D. G. (2018). Turnover of tonoplast proteins. *Plant Physiol.* 177 10–11. 10.1104/pp.18.00322 29720533 PMC5933132

[B28] Jaillais Y. Fobis-Loisy I. Miege C. Rollin C. Gaude T. (2006). AtSNX1 defines an endosome for auxin-carrier trafficking in *Arabidopsis*. *Nature* 443 106–109. 10.1038/nature05046 16936718

[B29] Jian W. Zhang D. W. Zhu F. Wang S. X. Pu X. J. Deng X. G. (2016). Alternative oxidase pathway is involved in the exogenous SNP-elevated tolerance of *Medicago truncatula* to salt stress. *J. Plant Physiol.* 193 79–87. 10.1016/j.jplph.2016.01.018 26962709

[B30] Khedr A. H. A. Abbas M. A. Wahid A. A. A. Quick W. P. Abogadallah G. M. (2003). Proline induces the expression of salt-stress-responsive proteins and may improve the adaptation of *Pancratium maritimum* L. to salt-stress. *J. Exp. Bot.* 54 2553–2562. 10.1093/jxb/erg277 14512386

[B31] Kleine-Vehn J. Leitner J. Zwiewka M. Sauer M. Abas L. Luschnig C. (2008). Differential degradation of PIN2 auxin efflux carrier by retromerdependent vacuolar targeting. *Proc. Natl. Acad. Sci. U.S.A.* 105 17812–17817. 10.1073/pnas.0808073105 19004783 PMC2584678

[B32] Lin H. X. Yang Y. Q. Quan R. D. Mendoza I. Wu Y. S. Du W. M. (2009). Phosphorylation of SOS3-like calcium binding protein8 by SOS2 protein kinase stabilizes their protein complex and regulates salt tolerance in *Arabidopsis*. *Plant Cell* 21 1607–1619. 10.1105/tpc.109.066217 19448033 PMC2700523

[B33] Liu W. Li R. J. Han T. T. Cai W. Fu Z. W. Lu Y. T. (2015). Salt stress reduces root meristem size by nitric oxide-mediated modulation of auxin accumulation and signaling in *Arabidopsis*. *Plant Physiol.* 168 343–356. 10.1104/pp.15.00030 25818700 PMC4424022

[B34] Liu W. C. Li Y. H. Yuan H. M. Zhang B. L. Zhai S. Lu Y. T. (2017). WD40-REPEAT 5a functions in drought stress tolerance by regulating nitric oxide accumulation in *Arabidopsis*. *Plant Cell Environ.* 40 543–552. 10.1111/pce.12723 26825291

[B35] Liu W. C. Yuan H. M. Li Y. H. Lu Y. T. (2015). CKA2 functions in H2O2-induced apoptosis and high-temperature stress tolerance by regulating NO accumulation in yeast. *FEMS Yeast Res.* 15:fov051. 10.1093/femsyr/fov051 26100262

[B36] Lozano-Juste J. Leon J. (2010). Enhanced abscisic acid-mediated responses in nia1nia2noa1-2 triple mutant impaired in NIA/NR-and AtNOA1-dependent nitric oxide biosynthesis in *Arabidopsis*. *Plant Physiol.* 152 891–903. 10.1104/pp.109.148023 20007448 PMC2815865

[B37] Munns R. Tester M. (2008). Mechanisms of salinity tolerance. *Annu. Rev. Plant Biol.* 59 651–681. 10.1146/annurev.arplant.59.032607.092911 18444910

[B38] Niemes S. Langhans M. Viotti C. Scheuring D. San Wan Yan M Jiang L. (2010). Retromer recycles vacuolar sorting receptors from the trans-Golgi network. *Plant J.* 61 107–121. 10.1111/j.1365-313X.2009.04034.x 19796370

[B39] Park H. J. Kim W. Y. Yun D. J. (2016). A new insight of salt stress signaling in plant. *Mol. Cells* 39 447–459. 10.14348/molcells.2016.0083 27239814 PMC4916396

[B40] Raghavendra A. S. Gonugunta V. K. Christmann A. Grill E. (2010). ABA perception and signalling. *Trends Plant Sci.* 15 395–401. 10.1016/j.tplants.2010.04.006 20493758

[B41] Ryu H. Cho Y. G. (2015). Plant hormones in salt stress tolerance. *J. Plant Biol.* 58 147–155. 10.1007/s12374-015-0103-z

[B42] Salanenka Y. Verstraeten I. Löfke C. Tabata K. Naramoto S. Glanc M. (2018). Gibberellin DELLA signaling targets the retromer complex to redirect protein trafficking to the plasma membrane. *Proc. Natl. Acad. Sci. U.S.A.* 115 3716–3721. 10.1073/pnas.1721760115 29463731 PMC5889667

[B43] Shi H. T. Li R. J. Cai W. Liu W. Wang C. L. Lu Y. T. (2012). Increasing nitric oxide content in *Arabidopsis thaliana* by expressing rat neuronal nitric oxide synthase resulted in enhanced stress tolerance. *Plant Cell Physiol.* 53 344–357. 10.1093/pcp/pcr181 22186181

[B44] Shi H. T. Liu W. Wei Y. X. Ye T. T. (2017). Integration of auxin/indole-3-acetic acid 17 and RGA-LIKE3 confers salt stress resistance through stabilization by nitric oxide in *Arabidopsis*. *J. Exp. Bot.* 68 1239–1249. 10.1093/jxb/erw508 28158805

[B45] Shi Q. H. Ding F. Wang X. F. Wei M. (2007). Exogenous nitric oxide protect cucumber roots against oxidative stress induced by salt stress. *Plant Physiol. Biochem.* 45 542–550. 10.1016/j.plaphy.2007.05.005 17606379

[B46] Stierhof Y.-D. Viotti C. Scheuring D. Sturm S. Robinson D. G. (2013). Sorting nexins 1 and 2a locate mainly to the TGN. *Protoplasma* 250 235–240. 10.1007/s00709-012-0399-1 22447127

[B47] Wilson I. D. Neill S. J. Hancock J. T. (2008). Nitric oxide synthesis and signalling in plants. *Plant Cell Environ.* 31 622–631. 10.1111/j.1365-3040.2007.01761.x 18034772

[B48] Xie Y. J. Mao Y. Lai D. W. Zhang W. Zheng T. Q. Shen W. B. (2013). Roles of NIA/NR/NOA1-dependent nitric oxide production and HY1 expression in the modulation of *Arabidopsis* salt tolerance. *J. Exp. Bot.* 64 3045–3060. 10.1093/jxb/ert149 23744476 PMC3741688

[B49] Xiong L. M. Schumaker K. S. Zhu J. K. (2002). Cell signaling during cold, drought, and salt stress. *Plant Cell* 14 S165–S183. 10.1105/tpc.000596 12045276 PMC151254

[B50] Yang Y. Q. Guo Y. (2018). Elucidating the molecular mechanisms mediating plant salt-stress responses. *New Phytol.* 217 523–539. 10.1111/nph.14920 29205383

[B51] Yoshida T. Fujita Y. Sayama H. Kidokoro S. Maruyama K. Mizoi J. (2010). AREB1, AREB2, and ABF3 are master transcription factors that cooperatively regulate ABRE-dependent ABA signaling involved in drought stress tolerance and require ABA for full activation. *Plant J.* 61 672–685. 10.1111/j.1365-313X.2009.04092.x 19947981

[B52] Yuan H. M. Liu W. C. Lu Y. T. (2017). CATALASE2 coordinates SA-mediated repression of both auxin accumulation and ja biosynthesis in plant defenses. *Cell Host Microbe* 21 143–155. 10.1016/j.chom.2017.01.007 28182949

[B53] Zhao M. G. Tian Q. Y. Zhang W. H. (2007). Nitric oxide synthase-dependent nitric oxide production is associated with salt tolerance in *Arabidopsis*. *Plant Physiol.* 144 206–217. 10.1104/pp.107.096842 17351048 PMC1913813

[B54] Zhu J. Wang W. S. Ma D. Zhang L. Y. Ren F. Yuan T. T. (2016). A role for CK2 beta subunit 4 in the regulation of plant growth, cadmium accumulation and H2O2 content under cadmium stress in *Arabidopsis thaliana*. *Plant Physiol. Biochem.* 109 240–247. 10.1016/j.plaphy.2016.10.004 27750098

