# Supplementary material for: SORTING NEXIN 1 Functions in Plant Salt Stress Tolerance Through Changes of NO Accumulation by Regulating NO Synthase-Like Activity
Source: Front Plant Sci. 2018 Nov 6;9:1634. doi: 10.3389/fpls.2018.01634 (PMC6277890; doi:10.3389/fpls.2018.01634)
Supplement: Supplementary file 1 [file Table_1.DOC]

**Supplemental Material**

**
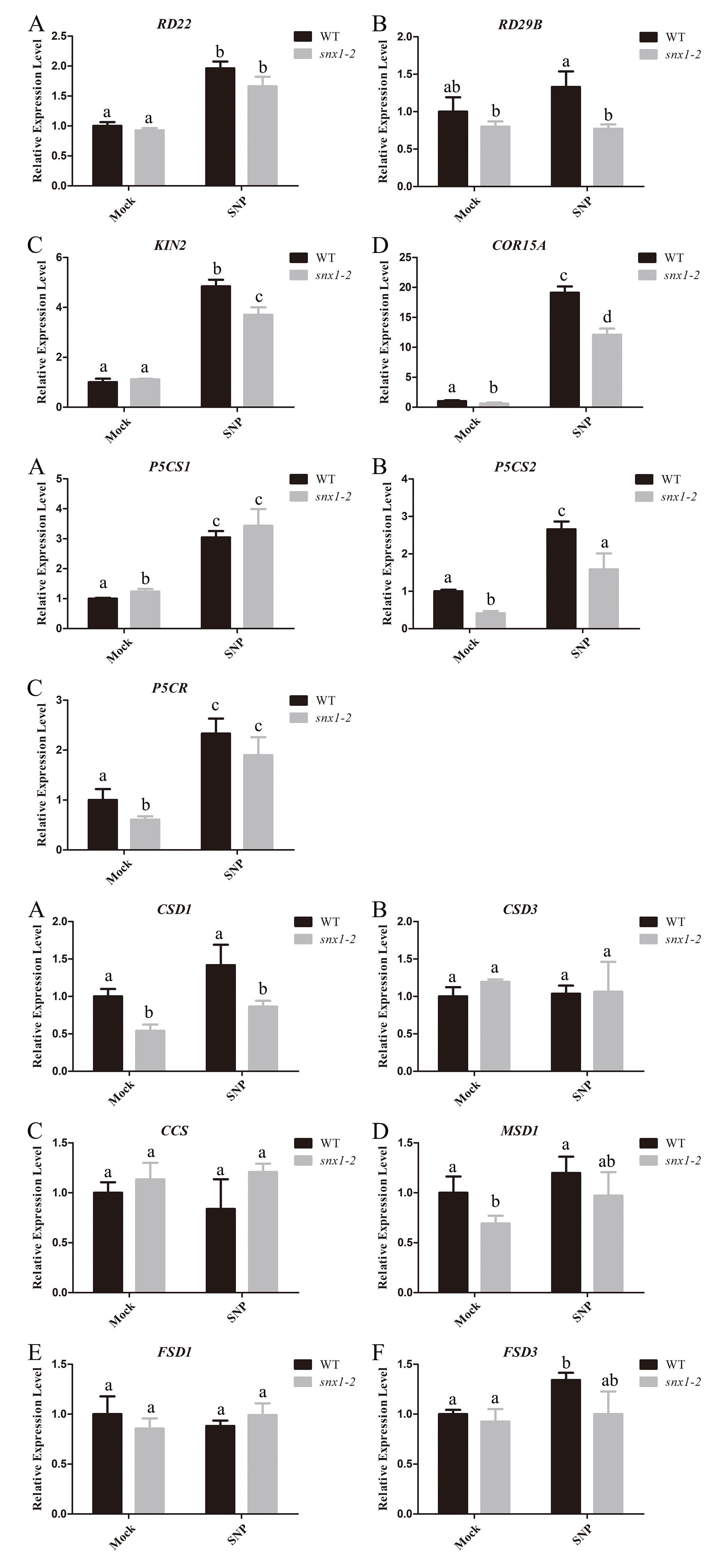
**

**Supplemental Figure S1. Expression of salt-responsive genes in the wild type and *snx1-2* plants treated with or without SNP.**

(A-D) 5-day-old wild-type and *snx1-2* mutant seedlings were treated with or without 5 μM SNP for 12 hours, and then assayed the expression of salt-responsive genes *RD22* (A), *RD29B* (B), *KIN2* (C), *COR15A* (D). Data shown are means ± SEM. Different letters indicate significant differences between treatments (*P* < 0.05 by one-way ANOVA with Tukey’s multiple comparison test).

**
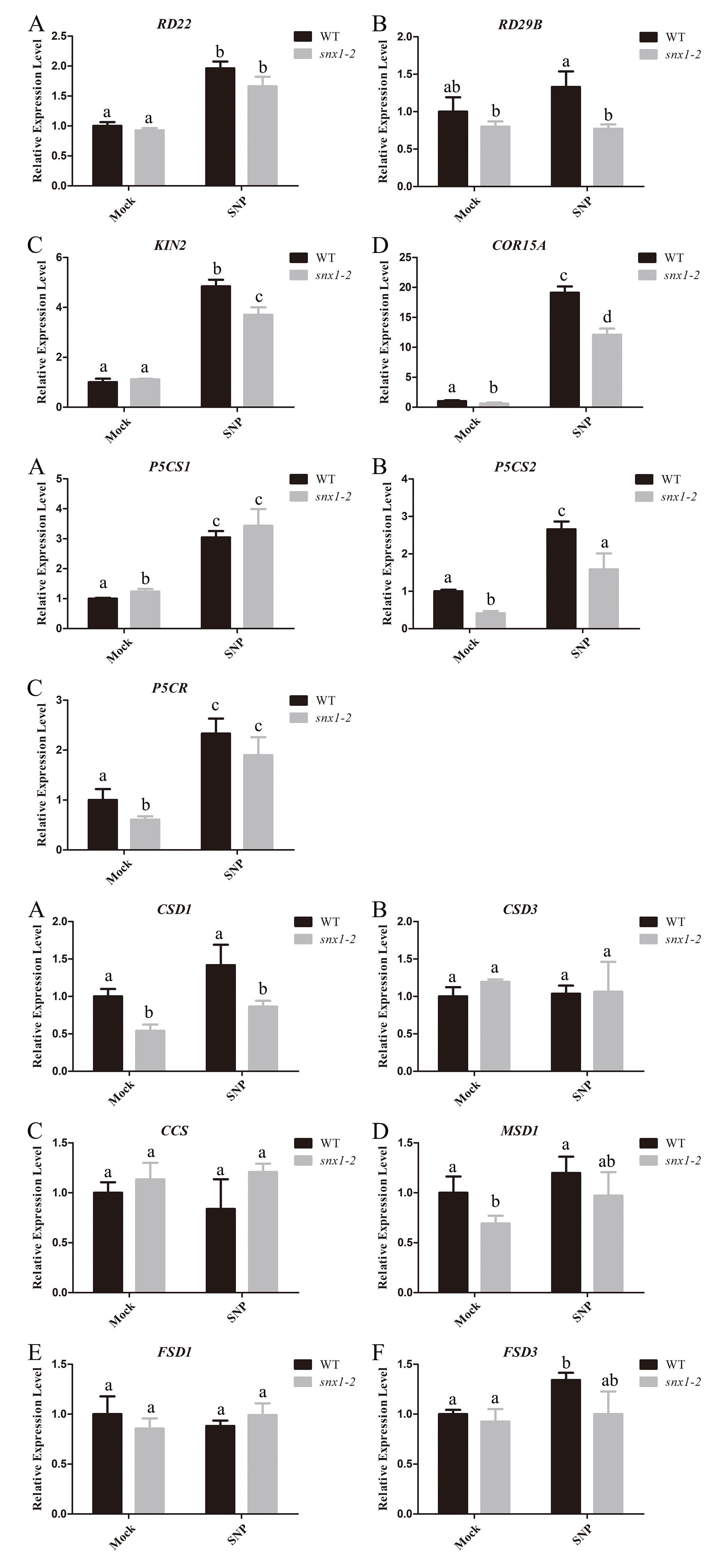
**

**Supplemental Figure S2. Expression of proline synthetic genes in the wild type and *snx1-2* plants treated with or without SNP.**

(A-C) 5-day-old wild-type and *snx1-2* mutant seedlings were treated with or without 5 μM SNP for 12 hours, and then assayed the expression of salt-responsive genes *P5CS1* (A), *P5CS2* (B), and *P5CR* (C). Data shown are means ± SEM. Different letters indicate significant differences between treatments (*P* < 0.05 by one-way ANOVA with Tukey’s multiple comparison test).

**
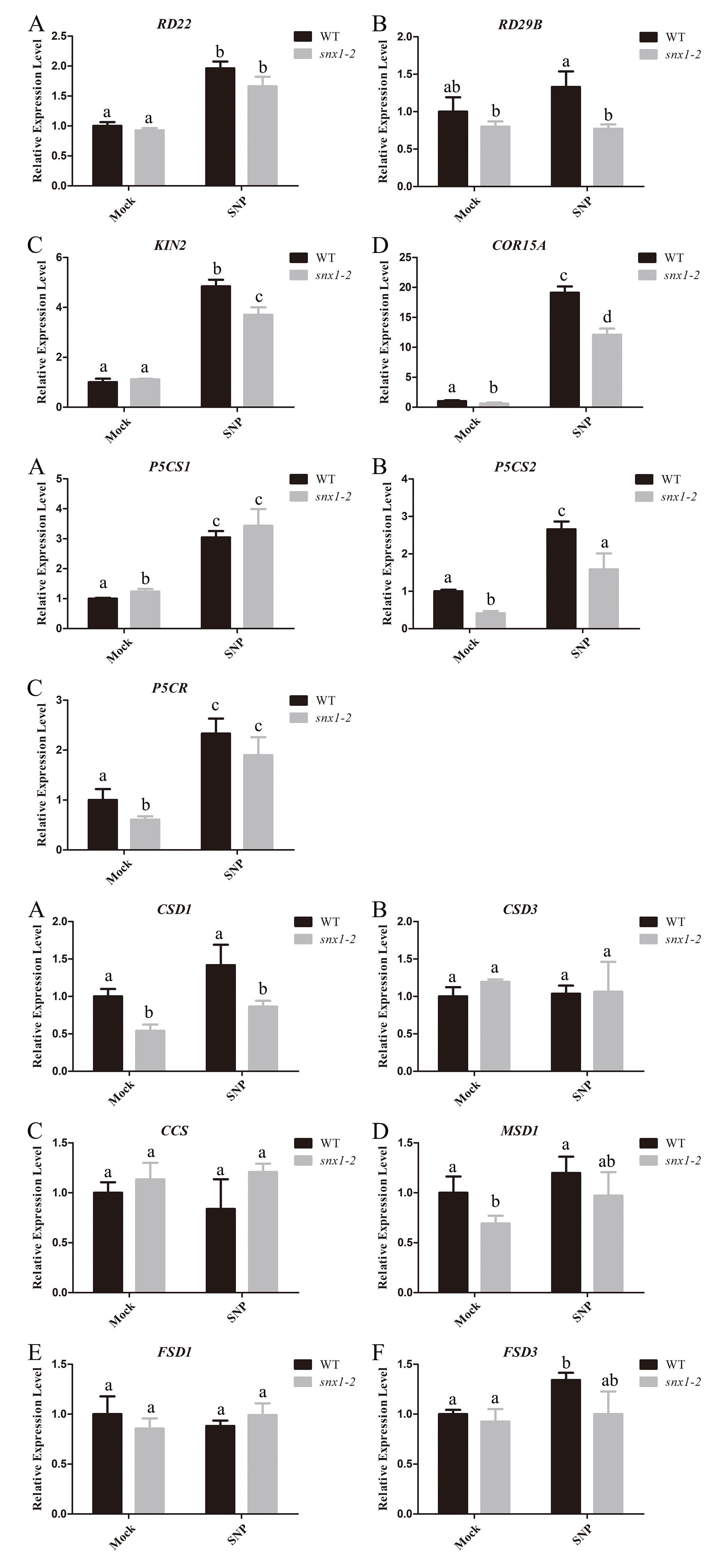
**

**Supplemental Figure S3. Expression of ROS-scavenging genes in the wild type and *snx1-2* plants treated with or without SNP.**

(A-F) 5-day-old wild-type and *snx1-2* mutant seedlings were treated with or without 5 μM SNP for 12 hours, and then assayed the expression of ROS-scavenging genes was assayed. Data shown are means ± SEM. Different letters indicate significant differences between treatments (*P* < 0.05 by one-way ANOVA with Tukey’s multiple comparison test).

**Supplementary table S1** List of the primers used in this study.

**Primer Name Sequence (5’ to 3’)**

Primers used for molecular cloning

*SNX1-F* CATGCCATGGGAGAGCACGGAGCAGCCG

*SNX1*-R CATGCCATGGTTAGACAGAATAAGAAGC

*SNX1-flagF* TCGGTACCAGTCCgGATCGAGAGCACGGAGCAGCCG

*SNX1-flagR* GTCGACTCTAGAGGATCcTTAGACAGAATAAGAAGC

Primers used for identification of genomic DNA PCR

LBb1.3 ATTTTGCCGATTTCGGAAC

LP TCAAGCACCCAAAAGCATTAC

RP TGGACAGATTCAGGTTTCAGG

Primers used for qRT-PCR

*SNX1-qF* TCTTCCAGAGAAGAGTGCTGTAGA

*SNX1-qR* CTCAGGGTGTAATGCTATTCGAT

*ACT2/8-q*F TAACAGGGAGAAGATGACTCAGATCA

*ACT2/8-q*R AAGATCAAGACGAAGGATAGCATGAG

*COR15a-q*F CTTACCTAATCAGTTAATTTCAAGCA

*COR15a-q*R TTAAACATGAAGAGAGAGGATATGG

*KIN2-qF* AATGTTCTGCTGGACAAGGC

*KIN2-q*R AACTCCCAAAGTTGACTCGGA

*CSD1-qF* CCAAAGGGGTTTCCTGAGA

*CSD1-qR* GCCTTCCTGGGTGAAAAAG

*CSD3-qF* TGGATTTCATATTCACTCTTTTGG

*CSD3-qR*  ATGGATTGAAGTGAGGTCCAG

*CCS-qF*  CAGGGAGCTTGTACAATCCAT

*CCS-qR*  GCCTCTAGTGTTCCCAGGTCT

*RD22-q*F TTCGCGGTGTTCTACTGCC

*RD22-q*R CGGAACCGCGTAGACGG

*RD29B-q*F GGCGGGCAAAGCGAG

*RD29B-q*R TGCCCGTAAGCAGTAACAGATC

*FSD1-qF* GAAATGTGCAGCTTCAATTACAA

*FSD1-qR*  TATGCGGCTCCAAAGCAT

*FSD3-qF*  GGATGGGTCTGGCTTGTCT

*FSD3-qR* CCACGCAGATGATTGGAATA

*MSD1-qF*  AGAGCGCCATCAAATTCAAC

*MSD1-qR*  TGGCTCTCCACCACCTTC

*P5CS1-qF* TGTGTGTTTGTGTATTTGGTTGAGAC

*P5CS1-qR* TGAGTACTAAGCAGAGAGGAAACAAAA

*P5CS2-qF*  CGAAAATCCCAGTGCTAGGC

*P5CS2-qR*  TGCCATGTCCAGTTTACCAGACT

*P5CR-qF* TTGGTGAGGCAGCTTCAGT

*P5CR-qR* CGCCAAACAACATAGCAACA
